# Supplementary material for: Retrospective Analysis of the Psychological Predictors of Public Health Support in Bulgarians at the Beginning of the Coronavirus Pandemic
Source: Brain Sci. 2023 May 19;13(5):821. doi: 10.3390/brainsci13050821 (PMC10216045; doi:10.3390/brainsci13050821)
Supplement: Supplementary file 1 [file brainsci-13-00821-s001.zip › File S1.pdf]

Research Methodology

Global survey by project “International Collaboration on the Social & Moral Psychology of COVID-19”

Authors: Jay Van Bavel, Mark Alfano, Paulo Sérgio Boggio, Valerio Capraro, Aleksandra Cichocka, Aleksandra Cisłak and Hallgeir Sjåstad

References:

Azevedo, Flavio, Tomislav Pavlović, Gabriel G. d. Rêgo, F. C. Ay, Biljana Gjoneska, Tom Etienne, Robert M. Ross, et al. 2022. Social and moral psychology of COVID-19 across 69 countries. *PsyArXiv*. Accessed March 27, 2023. https://doi.org/10.31234/osf.io/a3562

ICSMP. An International Collaboration on the Social & Moral Psychology of COVID-19. Home page. Accessed March 27, 2023. https://icsmp-covid19.netlify.app/index.html

COVID-19 Many Lab Study – Bulgaria

Physical contact

Be as accurate as you can:

During the days of the coronavirus (COVID-19) pandemic, I have been ...

Strongly disagree | **Neither agree nor disagree** | **Agree**

0 10 20 30 40 50 60 70 80 90 100

Staying at home as much as practically possible.	
Visiting friends, family, or colleagues outside my home.	
Keeping the number of grocery store visits at an absolute minimum.	
Keeping physical distance from all other people outside my home.	
Avoiding handshaking with people outside my home.	

Physical hygiene

Be as accurate as you can: During the days of the coronavirus (COVID-19) pandemic, I have been ...

	Strongly disagree	Neither agree nor disagree	Agree

	0	10	20	30	40	50	60	70	80	90	100
Washing my hands longer than usual.											
Washing my hands (with soap) more thoroughly than usual.											
Always washing my hands immediately after returning home.											
Disinfecting frequently used objects, such as mobile phones and keys.											
Sneezing and coughing into my upper sleeve.											

Anti-corona policy support

Be as accurate as you can: During the days of the coronavirus (COVID-19) pandemic, I have been ...

	Strongly disagree	Neither agree nor disagree	Agree

	0	10	20	30	40	50	60	70	80	90	100
In favor of closing all schools and universities.											
In favor of closing all bars and restaurants.											
In favor of closing all parks.											
In favor of forbidding all public gatherings where many people are gathered at one place (sports and culture).											
In favor of forbidding all non-necessary travel.											

End of Block: Anti-corona policy support

Psychological well-being

In general, to what extent do you feel happy these days?

	Very unhappy										Very happy
	0	1	2	3	4	5	6	7	8	9	10
1 ()											

Please imagine a ladder, with steps numbered 0 at the bottom and 10 at the top. The top represents the best possible life for you, and the bottom represents the worst possible life for you. On which step of the ladder would you say you personally feel you stand at this time?

	Worst possible life										Best possible life
	0	1	2	3	4	5	6	7	8	9	10
1 ()											

Collective narcissism

For each of the following statements, please select the answer that best describes whether you agree or disagree.

	Strongly disagree					Neither agree nor disagree					Strongly agree
	0	1	2	3	4	5	6	7	8	9	10
Bulgarians deserves special treatment.											
Not many people seem to fully understand the importance of Bulgarians.											
I will never be satisfied until Bulgarians gets the recognition it deserves.											

National Identification

For each of the following statements, please select the answer that best describes whether you agree or disagree.

	Strongly disagree					Neither agree nor disagree					Strongly agree
	0	1	2	3	4	5	6	7	8	9	10
I identify as Bulgarian.											
Being a Bulgarian is an important reflection of who I am.											

Conspiracy Theories COVID-19

For each of the following statements, please select the answer that best describes whether you agree or disagree.

	Strongly disagree					Neither agree nor disagree					Strongly agree
	0	1	2	3	4	5	6	7	8	9	10
The coronavirus (COVID-19) is a bioweapon engineered by scientists.											
The coronavirus (COVID-19) is a conspiracy to take away citizen's rights for good and establish an authoritarian government.											
The coronavirus (COVID-19) is a hoax invented by interest groups for financial gains.											
The coronavirus (COVID-19) was created as a cover up for the impending global economic crash.											

Open-mindedness

For each of the following statements, please select the answer that best describes whether you agree or disagree.

Strongly disagree	Neither agree nor disagree	Strongly agree

0	1	2	3	4	5	6	7	8	9	10

I think that paying attention to people who disagree with me is a waste of time.	
I feel no shame learning from someone who knows more than me.	
If I do not know much about some topic, I don't mind being taught about it, even if I know about other topics.	
Even when I have high status, I don't mind learning from others who have lower status.	
Only wimps admit that they've made mistakes.	
I don't take people seriously if they're very different from me.	

Morality-as-cooperation (short version)

When you decide whether something is right or wrong, to what extent are the following considerations relevant to your thinking?

Strongly disagree	Neither agree nor disagree	Strongly agree

0	1	2	3	4	5	6	7	8	9	10

Whether or not someone helped a member of their family.	
Whether or not someone worked to unite a community.	
Whether or not someone kept their promise.	
Whether or not someone showed courage in the face of adversity.	
Whether or not someone deferred to those in authority.	
Whether or not someone kept the best part for themselves.	
Whether or not someone kept something that didn't belong to them.	

Trait optimism

For each of the following statements, please select the answer that best describes whether you agree or disagree.

Strongly disagree | **Neither agree nor disagree** | **Strongly agree**

0 1 2 3 4 5 6 7 8 9 10

As a person, I am always optimistic for my future.	
Overall, I expect more good things to happen to me than bad.	

Social belonging

For each of the following statements, please select the answer that best describes whether you agree or disagree.

Strongly disagree | **Neither agree nor disagree** | **Strongly agree**

0 1 2 3 4 5 6 7 8 9 10

I feel connected with others.	
When I am with other people, I feel included.	
I feel accepted by others.	
I have close bonds with family and friends.	

Trait self-control

For each of the following statements, please select the answer that best describes whether you agree or disagree.

	Strongly disagree					Neither agree nor disagree					Strongly agree
	0	1	2	3	4	5	6	7	8	9	10
I am good at resisting temptation.											
I am able to work effectively toward long-term goals.											
I have a hard time breaking bad habits.											
I am lazy.											

Narcissism

For each of the following statements, please select the answer that best describes whether you agree or disagree.

	Strongly disagree					Neither agree nor disagree					Strongly agree
	0	1	2	3	4	5	6	7	8	9	10

I react annoyed if another person steals the show from me.	
I deserve to be seen as a great personality.	
I want my rivals to fail.	
Being a very special person gives me a lot of strength.	
I manage to be the center of attention with my outstanding contributions.	
Most people are somehow losers.	

Moral Identity

Listed below are some characteristics that might describe a person: caring, compassionate, fair, friendly, generous, helpful, hardworking, honest, kind. The person with these characteristics could be you or it could be someone else. For a moment, visualize in your mind the kind of person who has these characteristics. Imagine how that person would think, feel, and act. When you have a clear image of what this person would be like, answer the following questions.

Strongly disagree				Neither agree nor disagree				Strongly agree		
0	1	2	3	4	5	6	7	8	9	10

It would make me feel good to be a person who has these characteristics.	
Being someone who has these characteristics is an important part of who I am.	
I often wear clothes that identify me as having these characteristics.	
I would be ashamed to be a person who had these characteristics.	
The types of things I do in my spare time (e.g., hobbies) clearly identify me as having these characteristics.	
The kinds of books and magazines that I read identify me as having these characteristics.	
Having these characteristics is not really important to me.	
The fact that I have these characteristics is communicated to others by my membership in certain organizations.	
I am actively involved in activities that communicate to others that I have these characteristics.	
I strongly desire to have these characteristics.	

Risk perception

Please answer the following questions as accurately as possible:

	**0% = Impossible**	**50% = Neither likely nor unlikely**	**100% = Certain**

0 10 20 30 40 50 60 70 80 90 100

By April 30, 2021: How likely do you think it is that you will get infected by the Coronavirus (Covid-19)?	
By April 30, 2021: How likely do you think it is that the average person in Bulgaria will get infected by the Coronavirus (Covid-19)?	

Please answer the following questions:

What is your sex?

Male (1)

Female (2)

Other (3)

How old are you?

What is your current marital status?

Single (1)

In a relationship (2)

Married (3)

How many children do you have? If none, please type 0.

How would you describe your current employment status?

Employed full time (1)

Employed part-time (2)

Unemployed / Looking for work (3)

Student (4)

Retired (5)

Other (6)
