# Supplementary material for: Retrospective Analysis of the Psychological Predictors of Public Health Support in Bulgarians at the Beginning of the Coronavirus Pandemic
Source: Brain Sci. 2023 May 19;13(5):821. doi: 10.3390/brainsci13050821 (PMC10216045; doi:10.3390/brainsci13050821)
Supplement: Supplementary file 1 [file brainsci-13-00821-s001.zip › Table S1.pdf]

Table S1. Descriptive statistics

	PC	PH	ACPS	CN	PWB	CT	NI	MC	OM	TO	SB	TSC	N	MI	RP
Valid	725	723	724	728	731	727	727	728	727	732	727	726	724	709	717
Missing	8	10	9	5	2	6	6	5	6	1	6	7	9	24	16
M	20.12	21.02	19.56	8.15	6.19	9.73	8.23	24.65	26.03	7.91	15.92	14.03	15.48	40.23	6.07
Me	21.00	22.00	21.00	8.00	6.00	9.00	9.00	25.00	26.00	8.00	16.00	14.00	16.00	41.00	6.00
Mo	22	25	25	3	8	4	10	24	26	8	16	14	14	41	6
S.D.	3.81	4.05	5.08	3.45	1.74	4.83	1.89	3.07	3.02	1.70	2.74	2.79	4.73	7.72	1.86
Skew	-1.057	-1.343	-1.130	.057	-.126	.431	-1.157	-.787	-1.150	-1.180	-1.022	-.311	.028	-.728	.101
K	1.211	1.538	.669	-.950	-.932	-.972	.865	2.149	2.701	1.740	1.656	.030	-.447	.597	.107
Min	5	5	5	3	2	4	2	9	9	2	4	5	6	9	0
Max	25	25	25	15	10	20	10	33	30	10	20	20	30	55	10

*Note: Mean-M, Median-Me, Mode-Mo, Standart Deviation-S.D., Skewness-Skew, Kurtosis-K, Minimum value-Min, Maximum value-Max, Standart deviation-S.D., Physical contact-PC, Physical hygiene-PH, Anti-Corona Policy Support-ACPS, Collective narcissism-CN, Psychological Well-being-PWB, Conspiracy Theories COVID-19-CT, National Identification-NI, Morality-as-cooperation-MC, Open-mindedness-OM, Trait Optimism-TO, Social Belonging-SB, Trait Self-control-TSC, Narcissism-N, Moral Identity-MI, Risk Perception-RP.
